# Tomato breeding in the genomics era: insights from a SNP array

**DOI:** 10.1186/1471-2164-14-354

**Published:** 2013-05-27

**Authors:** Marcela Víquez-Zamora, Ben Vosman, Henri van de Geest, Arnaud Bovy, Richard GF Visser, Richard Finkers, Adriaan W van Heusden

**Affiliations:** 1 Wageningen UR Plant Breeding, P.O. Box 16, AJ, Wageningen, 6700, The Netherlands; 2 Centre for Biosystems Genomics, P.O. Box 98, AB, Wageningen, 6700, The Netherlands; 3 Bioscience, Plant Research International, P.O. Box 619, AP Wageningen, 6700, The Netherlands; 4 Graduate School Experimental Plant Sciences, Wageningen Campus, PB Wageningen, 6807, The Netherlands

**Keywords:** Single Nucleotide Polymorphisms (SNP), Custom made infinium array, Tomato wild relatives

## Abstract

**Background:**

The major bottle neck in genetic and linkage studies in tomato has been the lack of a sufficient number of molecular markers. This has radically changed with the application of next generation sequencing and high throughput genotyping. A set of 6000 SNPs was identified and 5528 of them were used to evaluate tomato germplasm at the level of species, varieties and segregating populations.

**Results:**

From the 5528 SNPs, 1980 originated from 454-sequencing, 3495 from Illumina Solexa sequencing and 53 were additional known markers. Genotyping different tomato samples allowed the evaluation of the level of heterozygosity and introgressions among commercial varieties. Cherry tomatoes were especially different from round/beefs in chromosomes 4, 5 and 12. We were able to identify a set of 750 unique markers distinguishing *S*. *lycopersicum* ‘Moneymaker’ from all its distantly related wild relatives. Clustering and neighbour joining analysis among varieties and species showed expected grouping patterns, with *S*. *pimpinellifolium* as the most closely related to commercial tomatoesearlier results.

**Conclusions:**

Our results show that a SNP search in only a few breeding lines already provides generally applicable markers in tomato and its wild relatives. It also shows that the Illumina bead array generated data are highly reproducible. Our SNPs can roughly be divided in two categories: SNPs of which both forms are present in the wild relatives and in domesticated tomatoes (originating from common ancestors) and SNPs unique for the domesticated tomato (originating from after the domestication event). The SNPs can be used for genotyping, identification of varieties, comparison of genetic and physical linkage maps and to confirm (phylogenetic) relations. In the SNPs used for the array there is hardly any overlap with the SolCAP array and it is strongly recommended to combine both SNP sets and to select a core collection of robust SNPs completely covering the entire tomato genome.

## Background

Landraces and wild relatives constitute a vast genetic resource that can be tapped to introduce novel traits into tomato breeding programmes [1]. During the last decades, the focus has mainly been on the introduction of disease resistance genes. But, within the breeding efforts, the lack of sufficient molecular markers in tomato has been a bottle neck in genetic and linkage studies. Although all known marker systems have been applied in tomato, most of them fall short in the genomics area mostly because they are too laborious and too low throughput [2]. These shortcomings are now being overcome by next generation sequencing projects and Single Nucleotide Polymorphisms (SNPs) identification [3]. The importance of SNPs as bi-allelic molecular markers is now widely recognized and their use is rapidly increasing [4,5] , since they have the advantage of being locus specific markers that can be scored co-dominantly in a flexible way. Technology has been developed for scoring single SNPs in thousands of different samples, all the way up to scoring millions of SNPs in a single sample [6].

Currently, the most widely used systems for high throughput SNP genotyping are the Illumina GoldenGate™, Infinium™ arrays and the KBioscience Competitive Allele‒Specific PCR genotyping system (KASPar: http://www.kbioscience.co.uk) [7-9]. The evolution of genotyping technologies has resulted in unprecedented possibilities for evaluating germplasm collections, characterizing populations, and finding markers linked to specific alleles of important genes. SNPs are also markers of choice for studying evolutionary processes [10]. Characterization of a large set of tomato varieties with a large number of markers can show the impact of breeding on the molecular level and the extent to which these markers are useful for variety identification [11,12].

A whole genome tomato genotyping array (custom made) using the Illumina® Infinium Beadarray technology [13] (http://www.illumina.com) was constructed to generate a multiplexing platform [7,14] to analyse tomato germplasm. A set of 5528 SNPs was used to evaluate more than a thousand tomato samples. This enabled us to compare data at the level of species, varieties and segregating populations. Within the Solanaceae Coordinated Agricultural Project (SolCAP: http://solcap.msu.edu/), in 2012 Sim *et al*. also developed a genotyping array [15]. However, they focused on different applications and, as we found out, with almost 100% different markers. We were interested in the question to what extent our SNP collection, which is based on a limited number of genotypes, can be applied for variety identification, phylogenetic analysis, genetic mapping, evaluation of introgressions and germplasm identification.

## Results

### SNPs evaluation and distribution

A set of 5528 SNP oligos (92%) passed the quality check of Illumina. From those oligos, 1980 originated from 454-sequencing, 3495 from Illumina Solexa and 53 from other studies (Table 1).

**Table 1:** Validated SNPs and their distribution over the chromosomes

**Chromosome**	**454**-**seq on cDNA from breeding lines**	**Illumina Solexa on gDNA from breeding lines**	**Illumina Solexa on gDNA from introgression free varieties**	**Markers from previous analysis**	**Total SNPs per chromosome**
1	195	45	103	4	347
2	183	38	47	6	274
3	94	10	136	0	240
4	244	149	359	8	760
5	138	460	97	2	697
6	375	349	183	2	909
7	106	54	43	0	203
8	87	28	30	9	154
9	299	151	32	12	494
10	33	36	519	0	588
11	104	57	41	3	205
12	107	249	225	6	587
Unknown position	15	26	28	1	70
Total	1980	1652	1843	53	5528

A full list of the SNPs can be found in Additional file 2: Table S1.

**Figure 1:**
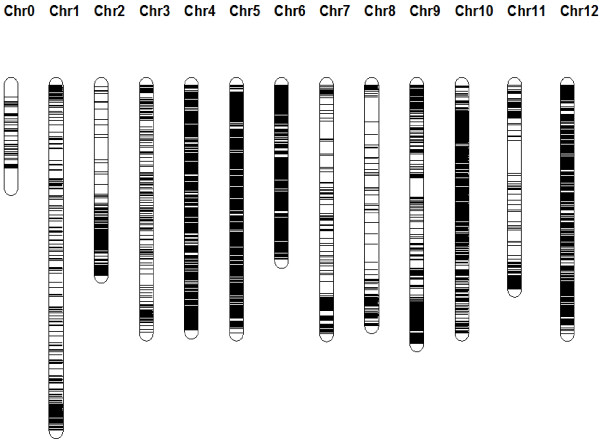
**Distribution of the SNP markers along the genome.** Physical positions according to the genome version in the SL2.30 version of the published tomato genome under the International Tomato Genome Sequencing Project [16].

As the SNPs were chosen before the tomato genome was publicly released (version 2.1) it was not completely clear how the markers would be distributed over the tomato chromosomes and what the marker density would be. Later all markers were assigned to their chromosomal position once the genome sequence (version SL2.30) was available and a good coverage and distribution of the markers over the physical map was observed (Figure 1). Some markers could not be placed on the genome and were placed on a pseudo molecule called Chromosome 0. On Chromosome 2, all markers are on the long arm because the short arm contains almost exclusively highly repetitive rDNA sequences [16]. Overall, the data quality was very good: The variety Heinz was used as control on each microtiter plate (12) and of the 66120 data points scored for this cultivar only 145 were deviating (0.2%) and in most cases this was due to no calls (NC).

However, approximately 10% of the markers could not be reliably scored mainly because of wrong automatic clustering by the GenomeStudio software. Closely linked markers in segregating populations can be used to find the correct score and the reasons for the mistakes in the automatic clustering (Additional file 1). Six percent of the SNPs resulted in NCs. These markers were removed resulting in 4072 SNPs for further analysis. Forty eight percent of the monomorphic markers within *S*. *lycopersicum* still were useful because they were polymorphic within tomato wild relatives or between wild relatives and cultivated tomato.

### Constructing genetic maps

**Figure 2:**
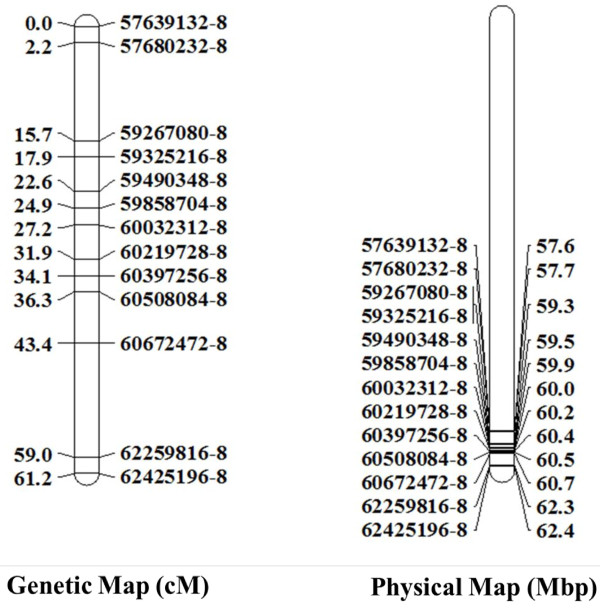
**Comparison of genetic linkage map (centimorgans) with a physical map (megabase pairs on the right) of chromosome 8 for an F2 population between two cherry type tomatoes.** Markers are indicated by the numbers ending on −8.

The SNPs were used to construct genetic maps in seven different mapping populations. For all of them the expected 12 linkage groups were found with most markers in the order as expected based on the tomato sequence (results not shown). Common ancestry of two parental lines resulted in regions without polymorphisms. Figure 2 shows an example of a linkage group created from the few SNPs showing recombination. On Chromosome 8 of an F2 population between two cherry tomato breeding lines only 13 polymorphic markers were found. Although the genetic map still spanned 61.2 cM the physical map showed that only a small part of the chromosome is covered by the 13 markers, apparently this part of 5 Mbp has a high recombination frequency (Figure 2).

### Variation among varieties

**Figure 3:**
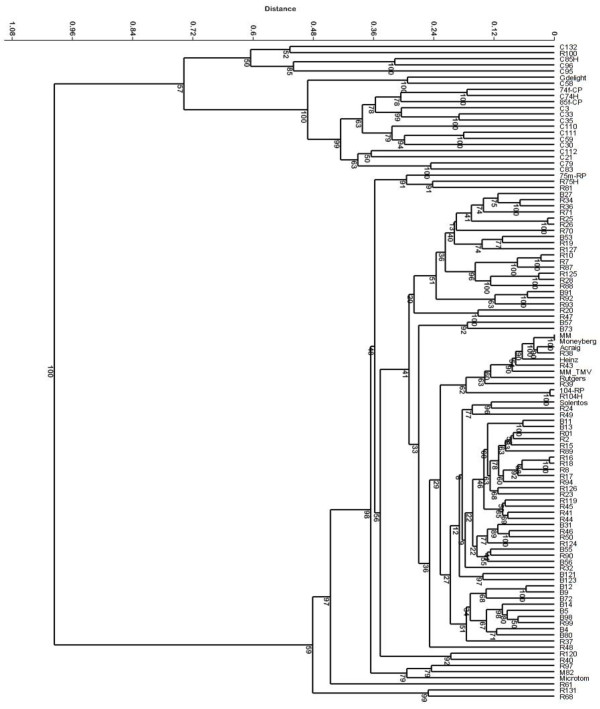
**Cluster analysis of commercial hybrids and tomato lines.** The Jukes-Cantor similarity measure with 1000 bootstraps was used.

**Figure 4:**
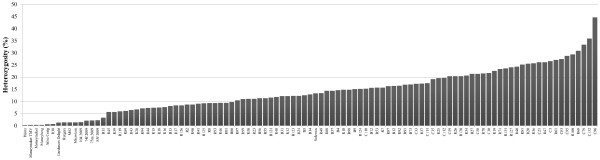
**Percentage of heterozygous markers (among 4072) found in hybrids, introgression free varieties and known commercial lines.**

**Figure 5:**
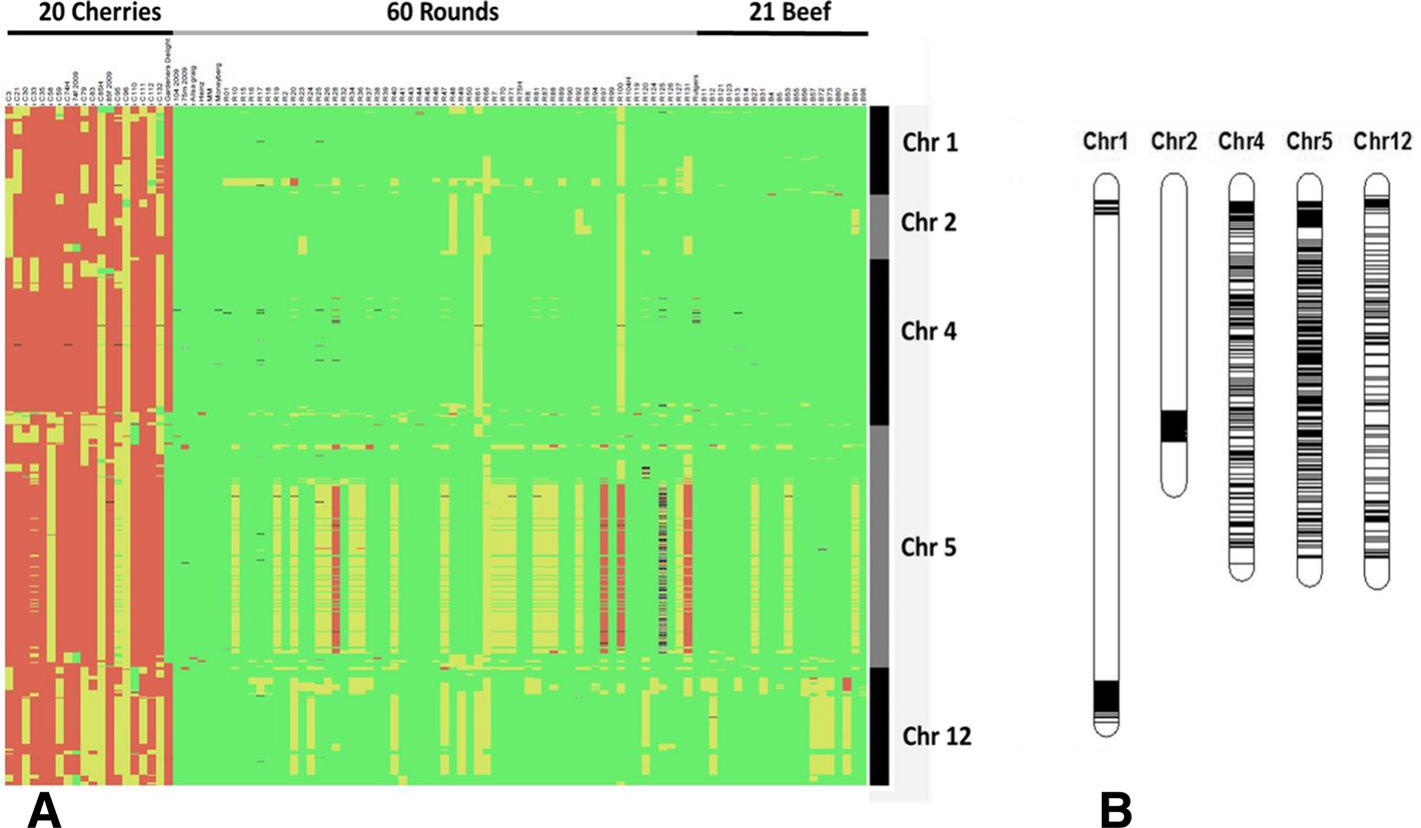
**Graphical representation of 955 SNPs distinguishing round**/**beef from cherry tomatoes. A**) Orange are cherry specific alleles, green round/beef alleles and yellow heterozygous calls (grey no-calls). **B**) Map-chart representation of the physical position of the 955 SNPs.

With the SNP markers we analysed 93 varieties plus some introgression free and other reference varieties (Additional file 2: Table S1). All varieties could be distinguished, although some were almost identical (Figure 3; Additional file 3 with the genotyping data for all materials used). Only the varieties Moneymaker and Moneyberg were completely identical. The percentage informative SNPs differed between the varieties. When we compare Moneymaker and R38 we will found about 5% polymorphic SNPs, between Moneymaker and R68 this was about 37%. The differences do not necessarily correspond to only introgressions (results not shown). The overall level of heterozygous markers within the varieties ranged from zero to almost 45% (Figure 4). The varieties included round, beef, and cherry types. In the dendrogram based on the SNP markers (Figure 3) the cherry tomatoes were clearly separated from the round/beef group, which were intermingled. Only the hybrid R100, classified as round, was in the group of cherry type tomatoes. This variety turned out to be a plum type of tomato and was misclassified as round. To see which markers contributed most to the separation of round/beef and cherry, we selected the markers distinguishing at least 90% of the cherries from round and beef tomatoes. This resulted in a selection of 955 SNPs that covered small areas of chromosomes 1 and 2 and the major central parts of chromosomes 4, 5 and 12 (Figure 5). However, also on chromosomes 3 and 10 small groups of markers specific to cherry tomatoes were found (results not shown).

Among the cherry varieties, Gardeners Delight did not have the cherry specific Chromosome 5, and this variety has somewhat larger fruits than what we considered as cherry. In total four round tomatoes clearly had the cherry specific Chromosome 5 (including R100), but after close inspection these were catalogued as deviating from round and more plum types.

### Identification of introgressions

**Figure 6:**
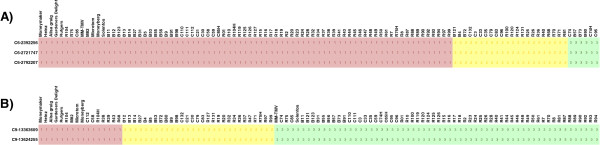
**Heat map comparison of markers in the regions of known introgressions: A) Root**-**knot nematode resistance (Mi.1**-**2 gene) and B) Tobacco mosaic virus resistance (tm2 gene).** Positions according to the ITAG2.30 ‘Release: genomic annotations’ (http://www.solgenomics.net). Introgression-free varieties in light red (1), heterozygous introgressions in yellow (2) and homozygous introgression in green (3).

Modern commercial varieties contain several introgressions from wild relatives. Most of these introgressions contain resistance genes [17]. We analysed a subset of varieties with known introgressions in detail and compared them with introgression-free varieties (Additional file 2: Table S1). Markers directly linked to known resistance-genes were selected and evaluated. Three markers were used on chromosome 6, two linked markers and one marker within the dominant Mi-1.2 gene (ITAG2.3 ‘Release: genomic annotations’ at http://www.solgenomics.net), conferring root-knot nematode resistance. In 79 varieties the genotype was identical to the introgression-free genotypes and 28 varieties had an introgression in this region (Figure 6). In 6 varieties the introgression was homozygous and in the 22 others heterozygous. Tobacco Mosaic Virus resistance (tm2 gene) is located on chromosome 9 and two markers were selected for this introgression, one corresponding to the gene and one to in the flanking region to confirm the introgression pattern. Figure 6 (and Additional file 4) shows that the region containing the *tm2* gene was present in 91 varieties taking into account the heterozygous introgressions. One of those varieties was R75, for which the introgression was not reported (Additional file 2: Table S3). There were 16 varieties that lacked the introgression. Differences in size of the introgressions were observed based on the polymorphism frequency among varieties. These differences were also compared with varieties annotated as introgression free and corroborated with known introgression (Additional file 2).

### SNPs for interspecific crosses in tomato

**Figure 7:**
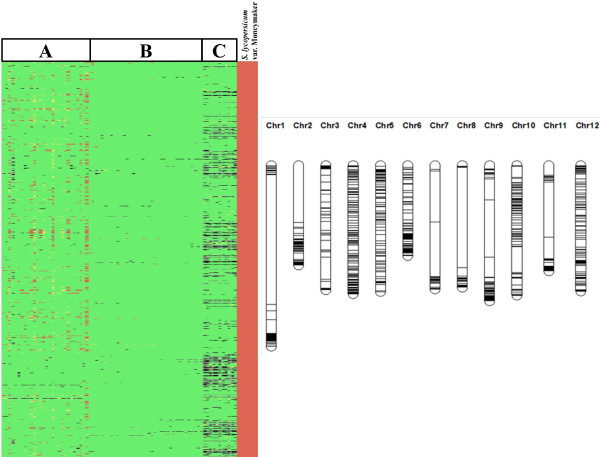
**Heat map and distribution of 750 SNPs over the twelve chromosomes differentiating *S. lycopersicum *cv. Moneymaker and wild relatives.** Genotype-calls: Red=[AA]; Yellow=[AB]; Green=[BB] and Black=no-call. **A**: 28 accessions from *S*. *pimpinellifolium*, **B**: 23 *S*. *cheesmaniae* and 14 *S*. *galapagense*. **C**: 2 *S*. *habrochaites*, 1 S.*chmielewskii*, 2 *S*. *neorickii*, 1 *S*. *pennellii*, 2 *S*. *arcanum* and 3 *S*. *chilense*.

*Solanum lycopersicum* cv. Moneymaker is the standard introgression free tomato used in our group to make mapping populations for breeding and genetic analysis. Therefore, we looked for markers that differentiate this *S*. *lycopersicum* cultivar from the majority of wild species accessions (Additional file 2: Table S2). A selection of 750 SNPs was polymorphic between Moneymaker and all screened accessions of the more distantly related wild relatives of tomato (*S*. *habrochaites* (2), *S*. *chmielewskii* (1), *S*. *neorickii* (2), *S*. *pennellii* (1), *S*. *arcanum* (2) and *S*. *chilense* (3)). Within this selection of markers, there were occasional (0.1%) non-polymorphisms with one or more of the 37 accessions of *S*. *cheesmaniae* and *S*. *galapagense* and a slight higher number of non-polymorphic cases (4%) within the 28 *S*. *pimpinellifolium* accessions (Figure 7).

### SNPs among tomato species

**Figure 8:**
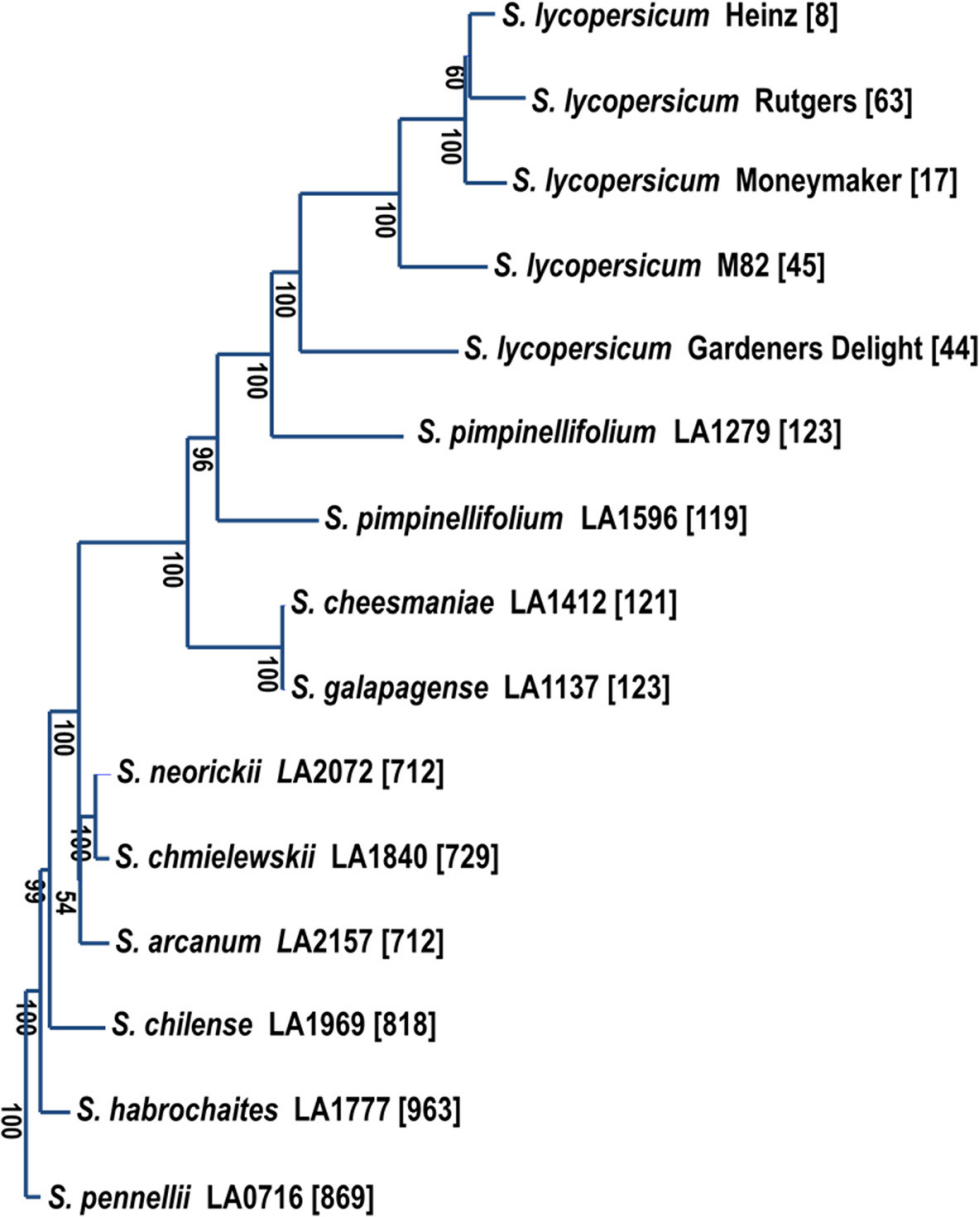
**Neighbour joining analysis of representative samples of tomato species using 4072 SNPs. *Solanum pennellii *accession LA0716 was used as outgroup.** Numbers at the nodes are bootstrap values for 1000 re-samplings. Numbers in brackets are the number of non-calls per genotype. Markers that were for more than 98% monomorphic, or had more than 25% heterozygous scores or more than 20% no-calls in the commercial hybrids or more than 50% in the wild relatives were removed from the dataset leaving a total of 4072 markers.

Representative accessions of wild relatives of tomato were analysed with the SNP array to establish relationships in *Solanum* sect. *Lycopersicon*. The phenetic analysis was carried out using neighbour joining. The resulting tree is shown in Figure 8. These relations are in accordance with Rodriguez *et al*. [18]. A BioNJ tree [19] can be found as Additional file 5. Figure 8 also shows that the number of NCs is becoming larger with increasing distance between the cultivated tomato and the wild relatives.

Accessions from *S*. *cheesmaniae* and *S*. *galapagense* clustered together (Figure 8). In our study only approximately 30 SNPs (from 5528) were found between the accessions of *S*. *cheesmaniae* and *S*. *galapagense* in spite of clear phenotypical differences in leaf structure and trichomes [20].

## Discussion

### Quality of SNP data

The high reproducibility of the results for the 12 Heinz samples shows the robustness of the data obtained with the Infinium array. This was also evident from the comparison between cv Moneymaker and cv Moneyberg where the only differences were a few NCs. Although the data were of high quality, individual SNP calls can be wrong. Wrong calls can be recognized in dense genetic linkage maps of a species from which the sequence is known. We observed errors in 10% of the SNPs, when using the standard settings of the Illumina Genome Studio software. Such errors can be corrected manually or 10% of the SNPs can be deleted [8]. Since the amount of data is vast, enough data remained after deleting 10% of the SNPs. Reasons for errors can be DNA quality, presence of outliers (Additional file 1) within the germplasm and, in few cases, double signalling due to duplications in the genome.

### General applicability of the SNPs

Even though the SNPs were looked for in a limited number (4) of breeding lines of *S*. *lycopersicum* in combination with four introgression free varieties, they were polymorphic enough in the *Solanum* sect. *Lycopersicon* germplasm to discriminate varieties and species as well as to confirm phenetic relations. This implies that many of the SNPs originated from the time before domestication [21-23].

The *S*. *lycopersicum* specific markers must have evolved after this species separated from the others. These markers will be polymorphic in any interspecific cross (Figure 7). A relatively cheap, SNP array with a limited number (as few as 20 per chromosome) of well distributed markers will be an excellent tool for a first fast characterization of any new interspecific mapping population involving *S*. *lycopersicum*. Based on our results such an array can be easily developed.

Furthermore, it is interesting to note that so many SNPs were found among the four breeding lines. This result was quite unexpected as *S*. *lycopersicum* is considered as a species with little genetic variation [1].

### The use of SNPs to improve the tomato genome sequence

One application of the SNP array was to compare genetic with physical positions when working with mapping populations. On the genetic linkage maps most of the markers were in the expected order; identical to the order in the assembled tomato sequence [16]. This confirmed the accurateness of the assembled Tomato Genome.

Some unassigned markers could be mapped to specific chromosomal positions in one or more of the linkage maps that we produced (results not shown). Comparison of genetic linkage maps and the physical linkage map also pointed out a misassembly on the long arm of chromosome 12 (between 48.8 Mbp and 61.7 Mbp; Additional file 6) in version 2.4 of the tomato genome. Also the data published by Sim *et al*. [15] suggest a disruption of marker order in the same region (see their Figure 3), but the conclusion that this might be due to a misassembly was not drawn. Markers should be used to genetically validate and further improve *de novo* genome assemblies.

### Variety identification

Several DNA profiling techniques have been used for variety identification [24]. For tomato, one of the most extensive studies was done by Bredemeijer *et al*. in 2002 [12] using simple sequence repeats (SSRs). They showed that 90% of the more than 500 varieties that were genotyped had a unique SSR profile using 20 markers (on average this is less than 2 markers per chromosome and one chromosome was even without markers). The SNP array covered between 150 and 900 markers per chromosome and all varieties could be distinguished, except the varieties Moneyberg and Moneymaker. That these two showed identical profiles means that they are highly related, if not identical. Both have been registered by the International Union for the Protection of New Varieties of Plants in the National Listing in Great Britain (UPOV [25]: http://www.upov.int), so phenotypic differences must have been seen. Under the UPOV act of 1991 such varieties would likely be considered as essential derived varieties [26]. The SNP markers developed in our study will be very useful for establishing whether varieties are essentially derived from other varieties using the protocol developed for lettuce [27].

The trend to exploit genes from tomato wild relatives for specific traits enlarges the variation in cultivated tomato and the differences among varieties [17]. Such introgressions can easily be detected using the SNP array as we have shown for the Mi1.2 and TMV gene. When gene-specific (or closely linked) SNP markers are used, genotyping may substitute phenotypic assays even in variety registration as was demonstrated by Arens *et al*. in 2010 [28]. The markers also allowed us to determine the level of heterozygous markers in present day varieties, which varied between zero and almost 45%. It is interesting to see that the highest numbers are found for some of the plum/cherry tomato. This is most likely because they are hybrids between round and cherry tomatoes and the 955 cherry specific SNPs will contribute to a large number of heterozygous markers (Figures 4 and 5). The high throughput SNP marker determination can be carried out at relatively low cost and is less laborious than other methods used. Therefore it is likely that SNP markers will be the markers of choice for variety identification and registration in future. However it may be anticipated that the SNP arrays will soon be replaced by complete sequencing of varieties.

### Differences between round/beef and cherry tomatoes

Many of the polymorphisms located on chromosomes 4, 5 and 12 were between round/beef and cherry tomatoes. This suggests that regions on these chromosomes are essential to get the full cherry tomato phenotype and that there is selection for these regions in breeding programs for cherry tomatoes. The fact that whole chromosomes (4, 5 and 12) look to be involved is possibly due to suppression of recombination in the large pericentromeric regions [29,30]. This is not the case on Chromosome 1 where the cherry region is a hotspot of recombination as shown in a RIL population of *S*. *lycopersicum* and *S*. *pimpinellifolium* under study (unpublished observations by the authors

Cherry type tomatoes have more SNPs in common with *S*. *pimpinellifolium* accessions than the round/beef varieties indicating that cherry tomatoes are closer to this wild relative than round and beef commercial lines. The varieties chosen for SNP selection might have been the reason that so many cherry specific markers were found. The SolCAP array also revealed different patterns of genetic variation particularly for chromosomes 2, 4, 5, 6 and 11. For chromosome 4 and 5 this is probably also due to the cherry round differences we observed. In general, relatively little is known about genomic regions distinguishing cultivated tomato gene pools [31].

Some regions are known to contain genes/QTLs that are related to differences between cherry and round. For instance a QTL for fruit weight and soluble solids content, is found on chromosome 2, QTLs for yield, brix, fruit weight, fruit shape, colour and epidermal reticulation have been mapped on chromosome 4 [32]. Chromosome 5 is known to harbour QTLs for fruit colour and QTLs for viscosity traits related to total red yield and pH in chromosome 12 are known [33,34].

### SNPs in *Solanum* sect. *Lycopersicon*

Our SNP based phenetic trees were comparable to the ones made by Bretó *et al*. in 1993 using isozymes [35], Palmer & Zamir in 1982[36] and Spooner *et al*. in 1993 with chloroplast DNA [37], McClean & Hanson in 1986 with mitochondrial DNA [38], Miller & Tanksley [1] with genomic DNA, Marshall *et al*. in 2001 [39] with internal transcribed spacer (ITS) region of nuclear ribosomal DNA sequences and, also Alvarez *et al*. in 2001 with microsatellite markers [40]. Peralta *et al*. [23] performed the most extensive taxonomic study of tomato and its wild relatives and our results confirm their findings.

In our analysis we found *S*. *pimpinellifolium* as the closest wild relative to *S*. *lycopersicum*, which is similar to observations made by Grandillo et al. [41] and The Tomato Genome Consortium in 2012[16]. The cherry tomato is considered either as a domesticated group or as an admixture of *S*. *pimpinellifolium* and *S*. *lycopersicum*[42]. *S*. *cheesmaniae* and *S*. *galapagense* are also very closely related to the domesticated tomato. Introgressions in the cultivated germplasm can affect the similarity weight in the relationships between *S*. *pimpinellifolium*, *S*. *galapagense* and *S*. *cheesmaniae* on one hand and *S*. *lycopersicum* hybrids on the other hand. For phylogenetic studies it is important to define the initial germplasm and its characteristics. In the case of *S*. *habrochaites* and *S*. *pennellii* the increased number of NCs decreased the resolution.

### Prospects of SNP data in tomato

For our custom made array, the SNP selection was based on commercial breeding lines. Sim *et al*. [15,31] developed a large SNP genotyping array using commercial varieties. To evaluate if the same SNPs were present, the precise SNP positions from both arrays were compared (allowing a window of ± 3 base pairs). Only 98 SNPs, less than 2% of our SNPs were found in the exact same position or within the allowed window. This means that there is still a large number of SNPs to be discovered in tomato. For further comparisons among the two arrays we made the SNPs including the flanking sequences available at: http://www.plantbreeding.wur.nl/Publications/SNP/4072SNP-Sequences.xlsx.

## Conclusions

Our results show that an SNP search in only a few breeding lines permitted the development of markers generally applicable in tomato and its wild relatives and furthermore that the Illumina bead array generated highly reproducible data. Our SNPs can be roughly divided in two categories: SNPs of which both forms are present in the wild relatives and in domesticated tomatoes and SNPs unique for the domesticated tomato. The SNPs can be used for genotyping, identification of varieties, comparison of genetic and physical linkage maps and to confirm phylogenetic relations. There is hardly any overlap with the SolCAP array and we suggest to combine both SNP sets and to select a core collection of robust SNPs completely covering the tomato genome for the development of future arrays.

## Methods

### Plant material

Tomato germplasm was obtained from the collection of Wageningen UR Plant Breeding, The Netherlands: the Tomato Genetics Resource Center (TGRC) at University of California, Davis; the Centre for Genetic Resources (CGN), The Netherlands; and from the breeding companies Monsanto, RijkZwaan, Takii, Vilmorin & Cie (VCo), ENZA and Syngenta. The evaluated material included hybrid varieties of the project within the Centre of Biosystems Genomics (CBSG: http://www.cbsg.nl). Based on QTL model predictions, four breeding lines were chosen to obtain a large diversity in taste related characteristics [43]. A half diallel was made with the four breeding lines resulting in six segregating populations. The parents were C74 (cherry, orange), C85 (cherry, red), R75 (round, yellow), and R104 (round, red). Further material included landraces, hybrids, commercial varieties, accessions of tomato wild relatives and mapping populations (Additional file 2: Table S1). The genotyping results of the varieties with the used SNPs can be found in Additional file 3.

### DNA and RNA extraction

Genomic DNA from young leaflets was extracted following a CTAB based protocol [44,45] adjusted for high throughput isolation. Two young leaflets were ground with a Retsch 300 mm shaker (Retsch BV, Ochten, The Netherlands) using 1 ml micronic tubes (Micronic BV, Lelystad, The Netherlands). The DNA pellets were washed in 76% EtOH with 10mM NH_4_Ac before re-suspending the DNA in TE buffer.

Total RNA was isolated using TRIzol reagent [46] according to the manufacturer’s instructions (Roche, Switzerland) and finally treated with DNaseI (Invitrogen).

### SNPs identified through Roche/454-sequencing

Total RNA was isolated from the four chosen breeding lines (C74, C85, R75 and R104), and at Vertis Biotechnologie AG (Freising, Germany: http://www.vertis-biotech.com/) cDNA was made. The 454 Sequencing gave 1.3 ×10^6^ reads of a median length of 400 base pairs. The reads were aligned to the tomato genome (v2.10) and SNPs were called using QualitySNP^ng^[3] after being adapted for large numbers of reads [47]. After Tomato v2.30 was available the SNP positions were renamed based on this version.

### SNPs identified through illumina/solexa-sequencing

A potential risk with the four breeding lines was that primarily interspecific SNPs would be found due to introgressed regions originating from tomato wild relatives (Additional file 2: Table S3). To include additional intraspecific (*S*. *lycopersicum*) variation four introgression free varieties were also included in the Illumina/Solexa sequencing. To reduce the complexity, genomic DNA (gDNA) of the eight different samples (C74, C85, R75, R104 and the introgression free varieties, Ailsa Craig/round, Rutgers/beef and Gardeners Delight/cherry plus the reference line Heinz/round) was digested with restriction enzyme *Mbo*I (four cutter) and the 400–600 bp fraction was cut out of a 1.5% agarose gel and purified. Theoretically, this should result in a coverage of at least 23x per fragment. After Illumina sequencing 15 × 10^6^ fragments were blasted against the Heinz v2.10 contigs and compared. The Illumina reads of 72 basepairs were aligned with the software tool Bowtie (>95% similarity) [48]. After alignment SNPs were called with VarScan (variant detection in massively parallel sequencing data) [49]. All SNPs with a minimal coverage of three in a genotype were listed in Excell. A SNP was called when it was present in at least six reads in one genotype and six reads in another genotype.

### Allocation of SNPs

Putative SNPs and their flanking regions were blasted against the then available contig sequences of tomato (Tomato WGS contigs v2.10) in order to choose SNPs as dispersed over the genome, when possible at least one SNP per contig. Later the availability of the tomato genome sequence (Tomato WGS chromosomes v2.3) allowed us to assign the SNPs to their physical location. A total of 6000 SNPs with two times 50 bp flanking sequences of Heinz were used for designing the oligo’s for the Illumina beadarray [13]. After the oligo’s were synthesized, ~8% of them did not comply to the quality standards set by Illumina and were discarded leaving 5528 SNP markers per array.

### Illumina® infinium bead array analysis

*Solanum sp*. DNA samples with a concentration of 50 ng/μl were sent to ServiceXS, Leiden, The Netherlands, where 4μl was processed according to the Infinium HD Ultra Assay protocol [13] and used for hybridization onto the BeadChip [50].

### Genotyping data processing

All the SNPs were named after their position on the SL2.30 version of the tomato genome sequence published online by the International Tomato Genome Sequencing Project (http://solgenomics.net/). This version contains approximately 85% of the tomato genome sequence. The lacking sequences are mostly highly repetitive or heterochromatic regions [16].

The Genotyping Module 1.9.4 of the Illumina’s software GenomeStudio® V2011.1 software package was used to analyse the genotyping results under default settings. The software assigned allele calls (‘GeneCall’) according to the intensity signals obtained, resulting in a [AA], [BB], [AB] or a non-call for each SNP. Advanced assembling within each correspondent analysis was performed and manual inspection and adjustment were performed in order to optimize call rates in the case of questionable SNPs. In particular those cases, and based on the knowledge on segregation patterns within the material, clustering errors were identified and amended [51].

Before further analysis, markers that were more than 98% monomorphic, were removed, as well as markers with more than 25% heterozygosity in accessions or breeding lines. Finally, also markers with a large number of NCs were removed. For this two thresholds for the percentage NCs were used: more than 20% NCs among the commercial hybrids and/or more than 50% among wild relatives.

When specific populations were evaluated, synchronization of parental lines together with the corresponding offspring was performed. This means that, for each analysis alleles were sorted according to the parent lines and replaced by a specific allele designation (A or B) for each parent.

### Data analysis

For cluster analysis the genotype calls were converted into numerical values: [AA]=1, [AB]=2, [BB]=3. Cluster analyses were done using the Jukes-Cantor similarity measure with 1000 bootstraps. Neighbour joining analysis using the Manhattan similarity measure with an out-group rooting and 1000 bootstraps was performed using the statistical package PAST version 2.12 [52]. The BioNJ analysis was carried out using SplitsTree version 4.6 with 1000 bootstraps.

Data visualization heat maps were made in GeneMaths XT 2.12 (Applied Maths). Linkage maps were constructed using JoinMap® version 4.1 (Kyazma©) [53]. The default calculation parameters were adjusted to cope with the large number of markers. In the similarity thresholds the option ‘show individual pairs with a similarity larger than’ was decreased from 0.95 to 0.7. Recombination frequency was used as a grouping parameter and the linkage parameters were set to take all LOD values from 0 to 100. The ‘Show strong linkages with a rec. freq. larger/smaller than’ were set to 0.5/0. The number of maximum linkages to show per locus was set to 0. As algorithm we used the ML (Maximum Likelihood) mapping option, and within the map building, the spatial sampling thresholds were set one to 0.1 the first and the rest to 0. The ‘Number of map optimization rounds per sample’ was fixed to 1. Thereafter, linkage groups were compared with chromosomal distribution in the physical maps using MapChart 2.2 [54].

## Competing interests

The authors declare that they have no competing interests.

## Authors’ contributions

MVZ carried out the analysis of the SNP data and wrote the manuscript. BV carried out the experimental set up, participated in the data analysis and co-wrote the manuscript. HvdG carried out the SNP search and helped in the bioinformatics support. AB managed the sampling of the tomato material and assisted the data analysis. RF carried out quality controls and the initial data analysis. RGFV was involved in the writing of the manuscript. AWvH conceived the study, coordinated the experimental set-up, helped in the SNP search, and co-wrote the manuscript. All authors read and approved the final manuscript.

## Supplementary Material

Additional file 1 **Example of genotyping graphs in GenomeStudio®.** SNP marker within one population in which two different groups were clustered automatically by the program in one group (the heterozygous group) due to an outlier sample (NTC). The right grouping is in Figure 1B, this was confirmed by flanking markers in a segregating population. The red circle exemplifies an outlier sample. Click here for file

Additional file 2: Table S1 Varieties from *S*. *lycopersicum* used for comparisons (also used by van Berloo, 2008). **Table S2**: Table with the accessions used in analysis. **Table S3**: Table with the introgressions known to be present in the initial breeding lines. Click here for file

Additional file 3 **All SNP scores in the varieties from *S. lycopersicum *used for comparisons (also used by van Berloo, 2008).** Click here for file

Additional file 4 **Heat map representation of polymorphisms found in the TMV region of chromosome 9.***Solanum lycopersicum* allele - gray background), yellow heterozygous and homozygous wild relative allele – green background. (PPTX 536 kb) Click here for file

Additional file 5 BioNJ tree with 1000 bootstrap analysis showing an implicit relation of the available species according the different tomato groups. Click here for file

Additional file 6 **Heat map of the genotype call of 188 markers distributed along Chromosome 12 of 100 RILs (horizontal) from a cross between *S. lycopersicum *cv Moneymaker (red) and *S. pimpinellifolium *G1.1554 (green).** Heterozygous calls (yellow) and NCs (black) are also included. Certain loci marked for reference as: sequence name / position (Mbp). The positions were blasted towards the published tomato genome version 2.4 [16]. Click here for file
