# Supplementary figures and images for: Tomato breeding in the genomics era: insights from a SNP array
Source: BMC Genomics. 2013 May 27;14:354. doi: 10.1186/1471-2164-14-354 (PMC3680325; doi:10.1186/1471-2164-14-354)

## Slide 1
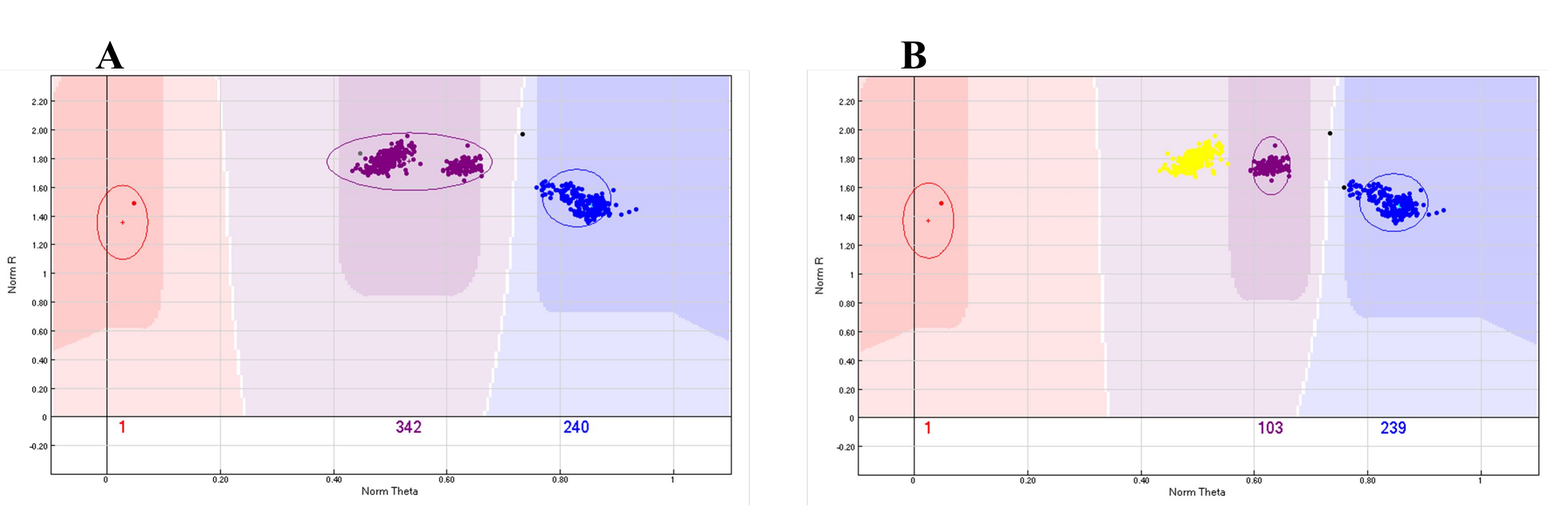

Supplement: Additional file 1 — Example of genotyping graphs in GenomeStudio®. SNP marker within one population in which two different groups were clustered automatically by the program in one group (the heterozygous group) due to an outlier sample (NTC). The right grouping is in Figure 1B, this was confirmed by flanking markers in a segregating population. The red circle exemplifies an outlier sample. [file 1471-2164-14-354-S1.pptx]

## Slide 1
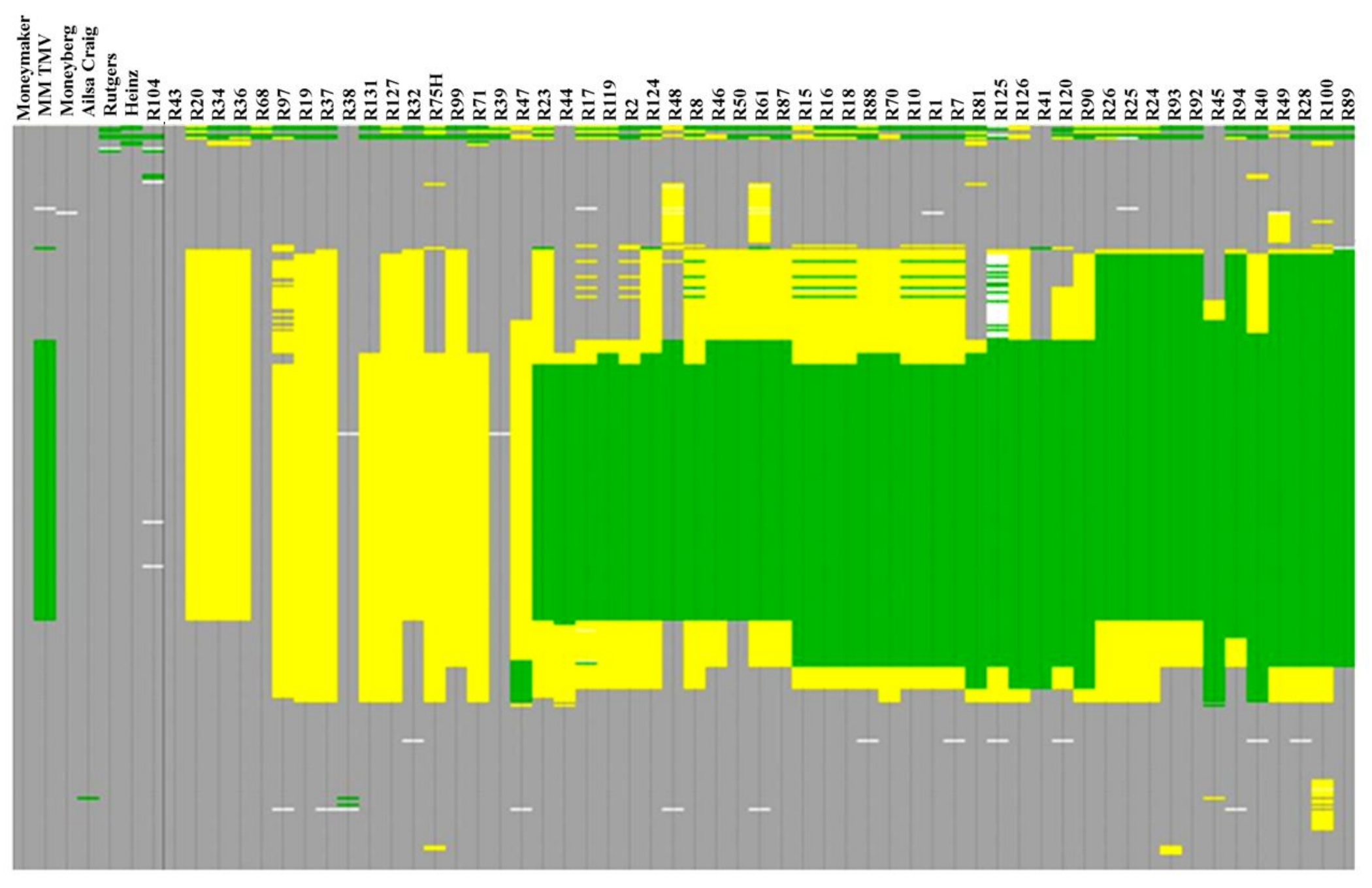

Supplement: Additional file 4 — Heat map representation of polymorphisms found in the TMV region of chromosome 9.Solanum lycopersicum allele - gray background), yellow heterozygous and homozygous wild relative allele – green background. (PPTX 536 kb) [file 1471-2164-14-354-S4.pptx]

## Slide 1
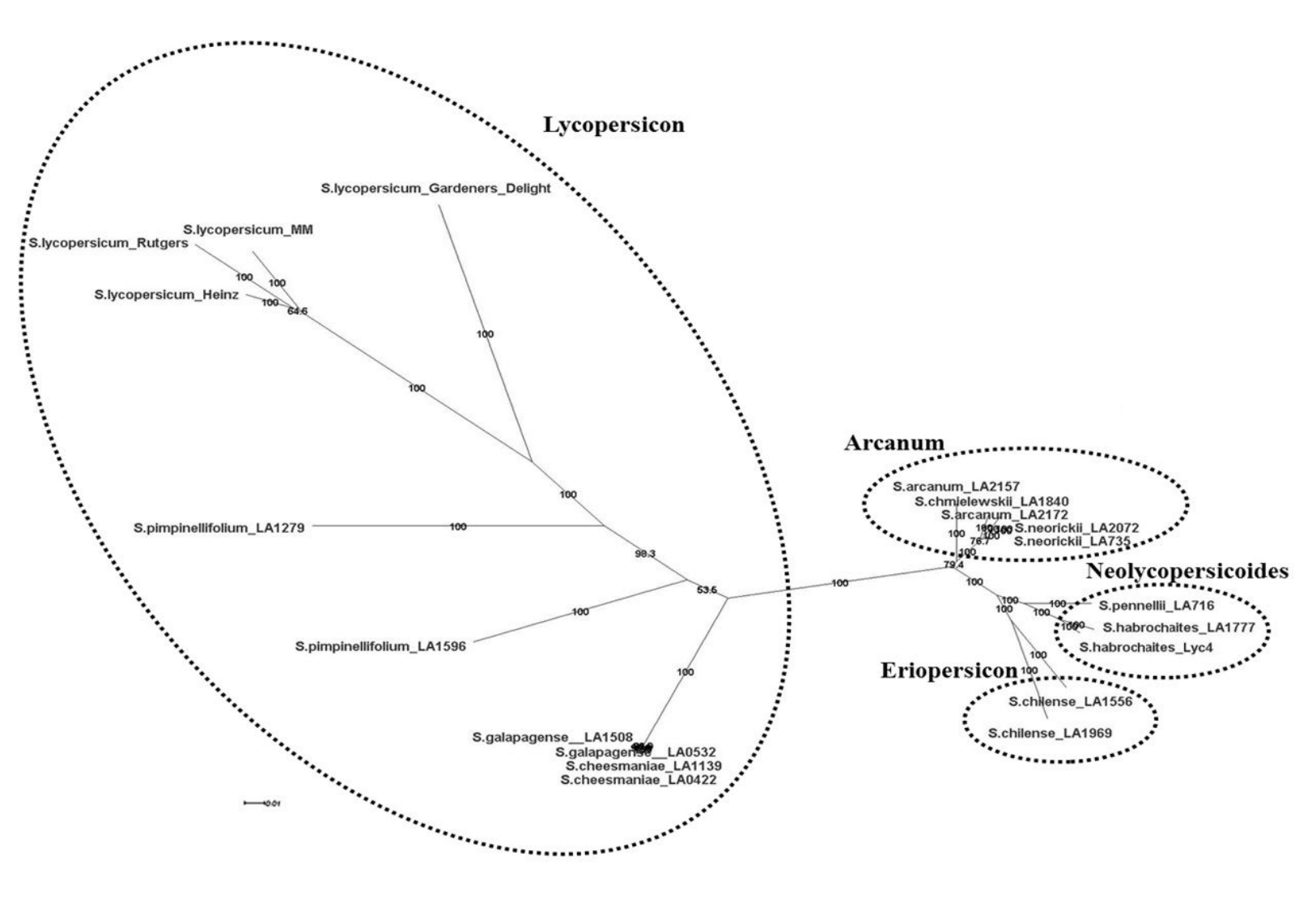

Supplement: Additional file 5 — BioNJ tree with 1000 bootstrap analysis showing an implicit relation of the available species according the different tomato groups. [file 1471-2164-14-354-S5.pptx]

## Slide 1
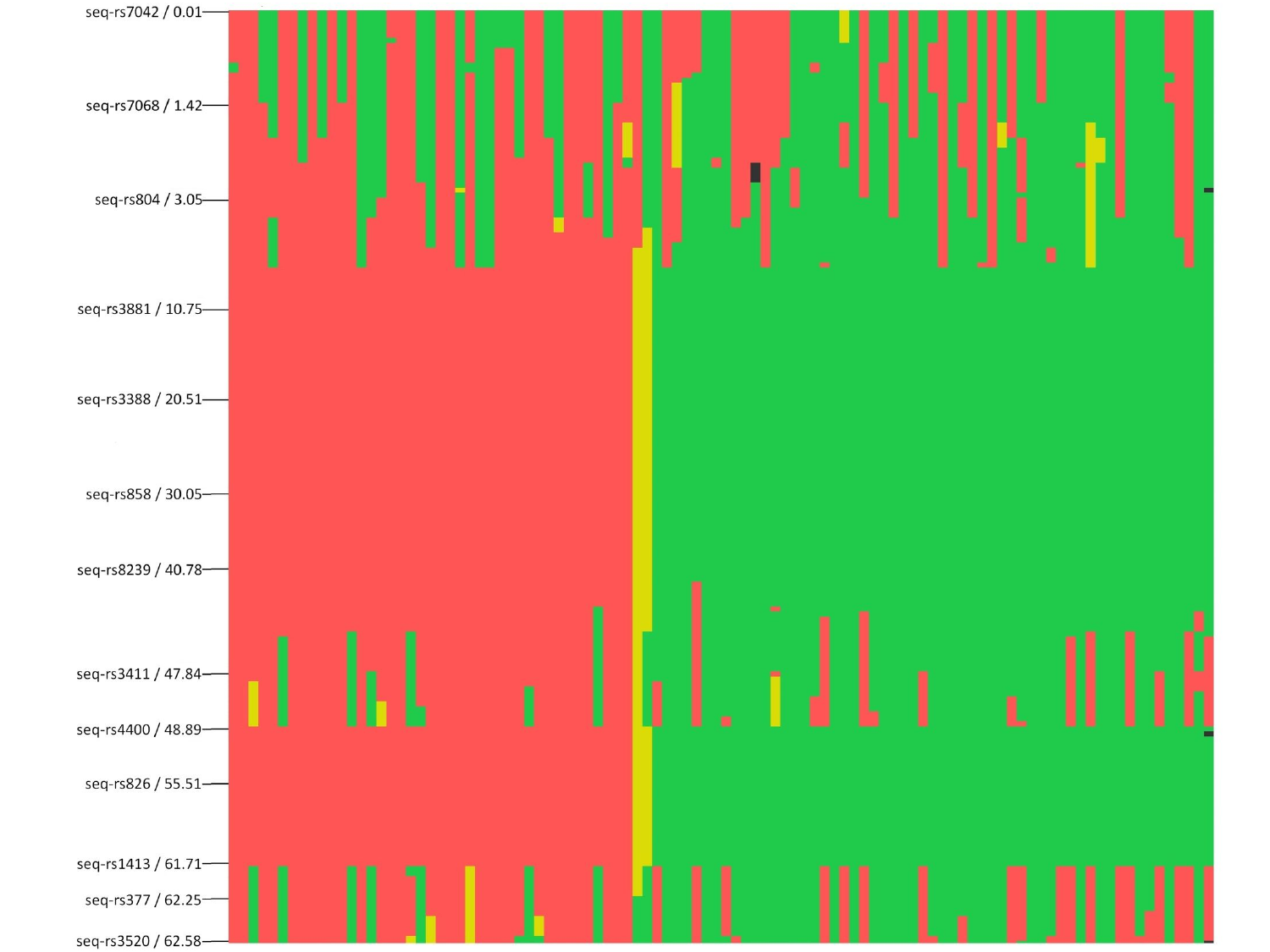

Supplement: Additional file 6 — Heat map of the genotype call of 188 markers distributed along Chromosome 12 of 100 RILs (horizontal) from a cross between S. lycopersicum cv Moneymaker (red) and S. pimpinellifolium G1.1554 (green). Heterozygous calls (yellow) and NCs (black) are also included. Certain loci marked for reference as: sequence name / position (Mbp). The positions were blasted towards the published tomato genome version 2.4 [16]. [file 1471-2164-14-354-S6.pptx]
