# Supplementary material for: Tomato breeding in the genomics era: insights from a SNP array
Source: BMC Genomics. 2013 May 27;14:354. doi: 10.1186/1471-2164-14-354 (PMC3680325; doi:10.1186/1471-2164-14-354)
Supplement: Additional file 2: Table S1 — Varieties from S. lycopersicum used for comparisons (also used by van Berloo, 2008). Table S2: Table with the accessions used in analysis. Table S3: Table with the introgressions known to be present in the initial breeding lines. [file 1471-2164-14-354-S2.docx]

**Additional Tables**

**Additional Table 1**. Varieties from *S. lycopersicum* used for comparisons (also used by van Berloo, 2008).

|  |  | | | | | |
| --- | --- | --- | --- | --- | --- | --- |
| *Introgression free varieties* | ***Reference varieties*** | ***Breeding lines*** | ***Varieties Cherry*** | ***Varieties Round*** | | ***Varieties Beef*** |
| Ailsa Craig | Heinz | C74 | C110 | R01 | R39 | B11 |
| Gardeners Delight | Moneymaker-TMV | C85 | C111 | R10 | R40 | B12 |
| Rutgers | Moneyberg | R75 | C112 | R100 | R41 | B121 |
| Moneymaker | Microtom | R104 | C132 | R104H | R43 | B123 |
|  | M82 |  | C21 | R119 | R44 | B13 |
|  | Solentos |  | C3 | R120 | R45 | B14 |
|  |  |  | C30 | R124 | R46 | B27 |
|  |  |  | C33 | R125 | R47 | B31 |
|  |  |  | C35 | R126 | R48 | B4 |
|  |  |  | C58 | R127 | R49 | B5 |
|  |  |  | C59 | R131 | R50 | B53 |
|  |  |  | C74H | R15 | R61 | B55 |
|  |  |  | C79 | R16 | R68 | B56 |
|  |  |  | C83 | R17 | R7 | B57 |
|  |  |  | C85H | R18 | R70 | B72 |
|  |  |  | C95 | R19 | R71 | B73 |
|  |  |  | C96 | R2 | R75H | B80 |
|  |  |  |  | R20 | R8 | B9 |
|  |  |  |  | R23 | R81 | B91 |
|  |  |  |  | R24 | R87 | B98 |
|  |  |  |  | R25 | R88 |  |
|  |  |  |  | R26 | R89 |  |
|  |  |  |  | R28 | R90 |  |
|  |  |  |  | R32 | R92 |  |
|  |  |  |  | R34 | R93 |  |
|  |  |  |  | R36 | R94 |  |
|  |  |  |  | R37 | R97 |  |
|  |  |  |  | R38 | R99 |  |

**Additional Table 2**. Accessions used in analysis

|  |  |  |  |
| --- | --- | --- | --- |
| *S. pimpinellifolium* accessions | *S. cheesmaniae* accessions | *S. galapagense* accessions | Further distant accessions |
| G1.1554 | LA0421 | LA0480A | *S. habrochaites* LA1777 |
| G1.1589 | LA0422 | LA0438 | *S. habrochaites* Lyc4 |
| LA1246 | LA0428 | LA0483 | *S. chmielewskii* LA1840 |
| LA1280 | LA0437 | LA0528 | *S. neorickii* LA2072 |
| LA1345 | LA0521 | LA0530 | *S. neorickii* LA735 |
| LA1349 | LA0522 | LA0532 | *S. pennellii*  LA716 |
| LA1355 | LA0524 | LA0748 | *S. arcanum* LA2172 |
| LA1374 | LA0529 | LA0929 | *S. arcanum* LA2157 |
| LA1472 | LA0746 | LA1137 | *S. chilense* LA1556 |
| LA1478 | LA0927 | LA1401 | *S. chilense*  LA1558 |
| LA1547 | LA0932 | LA1408 | *S. chilense* LA1969 |
| LA1577 | LA1035 | LA1452 |  |
| LA1580 | LA1039 | LA1508 |  |
| LA1584 | LA1040 | LA1627 |  |
| LA1596 | LA1041 |  |  |
| LA1599 | LA1042 |  |  |
| LA1601 | LA1043 |  |  |
| LA1611 | LA1139 |  |  |
| LA1645 | LA1404 |  |  |
| LA1660 | LA1409 |  |  |
| LA1670 | LA1412 |  |  |
| LA1719 | LA1447 |  |  |
| LA1924 | LA1450 |  |  |
| LA1936 |  |  |  |
| LA1993 |  |  |  |
| LA2097 |  |  |  |
| LA2533 |  |  |  |
| LA2854 |  |  |  |

**Additional Table 3**. Introgressions known to be present in the initial breeding lines.

| **Introgressions** | **Chr** | **C74** | **C85** | **R75** | **R104** | **Origin** |
| --- | --- | --- | --- | --- | --- | --- |
| Tomato Ripening-Inhibitor (Rin) | 5 |  |  |  |  | *S. lycopersicum* |
| Cladosporium 5 | 5 |  |  |  |  | *L. peruvianum* |
| TYLCV | 6 |  |  |  |  | *S. chilense / L. hirsutum* |
| Nematode | 6 |  |  |  |  | *L. peruvianum* |
| ToMV | 9 |  |  |  |  | *L. peruvianum* |
| ToMV (race 0,1,2) | 9 |  |  |  |  | *L. peruvianum* |
| Verticillium albo atrum | 9 |  |  |  |  | *S. lycopersicum var. cerasiforme* |
| Leaf mold (A,B,C,D,E) | 9 |  |  |  |  | *L. peruvianum* |
| Fusarium race 0 (ex1) | 11 |  |  |  |  | *S. pimpinellifolium / S. pennellii* |
| Fusarium crown and rootrot | ? |  |  |  |  | *L. peruvianum* |

**Additional Figures**

**Additional Figure 1.** Example of genotyping graphs in GenomeStudio^®^. SNP marker within one population in which two different groups were clustered automatically by the program in one group (the heterozygous group) due to an outlier sample (NTC). The right grouping is in figure 1B, this was confirmed by flanking markers in a segregating population. The red circle exemplifies an outlier sample.

**Additional Figure 2.** Heat map representation of polymorphisms found in the TMV region of chromosome 9. *Solanum lycopersicum* allele - gray background), yellow heterozygous and homozygous wild relative allele – green background.

**Additional Figure 3**. BioNJ tree with 1000 bootstrap analysis showing an implicit relation of the available species according the different tomato groups.

**Additional Figure 4**. Heat map of the genotype call of 188 markers distributed along Chromosome 12 of 100 RILs (horizontal) from a cross between *S. lycopersicum* cv Moneymaker (red) and *S. pimpinellifolium* G1.1554 (green). Heretozygous calls (yellow) and NCs (black) are also included. Certain loci marked for reference as: sequence name / position (Mbp). The positions were blasted towards the published tomato genome version 2.4 (The Tomato Genome Consortium, 2012).
